# Paucisymptomatic Tetralogy of Fallot diagnosed in a 56-year-old patient: a case report

**DOI:** 10.1186/s43044-023-00372-3

**Published:** 2023-05-26

**Authors:** Kaouthar Hakim, Rihab Benothman, Nouha Mekki, Hela Msaad, Fatma Ouarda

**Affiliations:** Pediatric Cardiology Department, La Rabta University Hospital of Tunis, Tunis El Manar University, Tunis, Tunisia

**Keywords:** Case report, Natural history, Late diagnosis, Tetralogy of Fallot

## Abstract

**Background:**

Tetralogy of Fallot (TOF) is the most common cyanotic congenital heart disease. It is generally diagnosed and surgically repaired early in life, with good overall outcomes.

**Case presentation:**

We report the case of a patient incidentally diagnosed with paucisymptomatic TOF at the age of 56 years old, during investigations for carbon monoxide poisoning. The patient had a history of thyroidectomy, arterial hypertension, and four uncomplicated vaginal deliveries.

**Conclusions:**

This case shows us that some patients with TOF can reach older ages without surgical correction. Late surgical repair should be meticulously decided on a case basis.

**Supplementary Information:**

The online version contains supplementary material available at 10.1186/s43044-023-00372-3.

## Background

Tetralogy of Fallot (TOF) is the most common cyanotic congenital heart disease (CHD), representing 7–10% of all CHD [1]. The surgical repair had markedly improved the survival of patients with TOF [2]. Only 3% of patients reached the age of 40 without surgical intervention [3]. Therefore, unrepaired TOF is very rarely diagnosed in adult patients. Herein, we report the case of a patient incidentally diagnosed with paucisymptomatic TOF at the age of 56 years old following carbon monoxide poisoning.

## Case presentation

Our patient was a 56-year-old female with a medical history of total thyroidectomy at the age of 33, arterial hypertension, and four uncomplicated vaginal deliveries. The patient had no family history of CHD. Her children were all in good health. In addition to thyroid hormone replacement therapy, she was treated with angiotensin-converting enzyme (ACE) inhibitors. The patient was admitted to a local hospital for carbon monoxide poisoning. She had a chest CT angiogram because she was oxygen-dependent. The CT angiography ruled out pulmonary embolism and COVID infection but revealed a right aortic arch, hypoplastic main and right pulmonary arteries, and a dilated left pulmonary artery. Therefore, she was referred to our department for further investigation.

The patient mentioned mild exertional dyspnea in the past few years. Clinical examination showed no clubbing or signs of heart failure, with a 91% oxygen saturation in room air. The patient was eupneic with normal breath sounds. The blood pressure was measured at 120/80 mmHg, and the heart rhythm was regular at 95 beats per minute. A systolic ejection murmur IV/VI was documented in the left second intercostal space on cardiac auscultation.

**Fig. 1:**
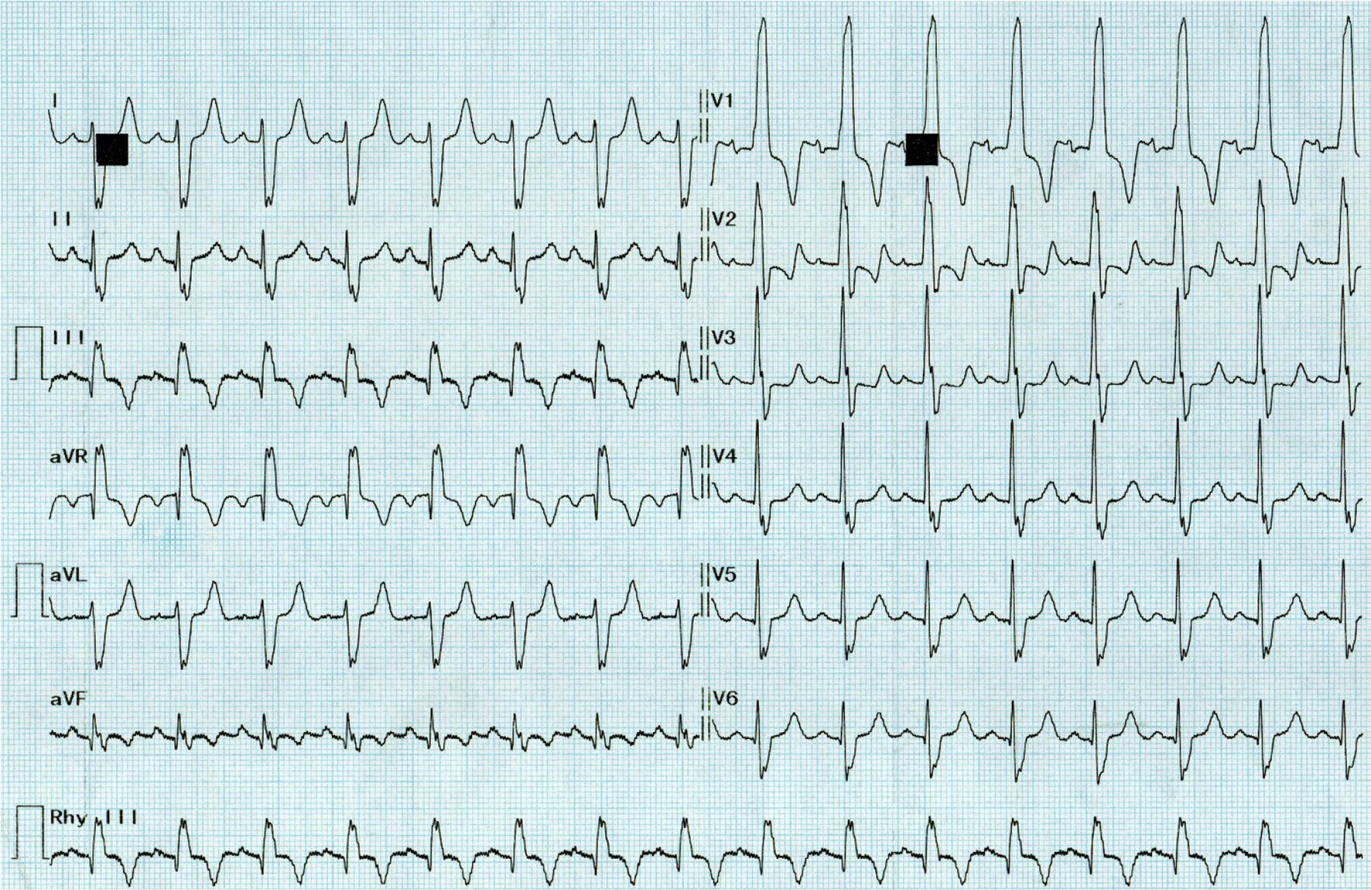
The electrocardiogram illustrates regular sinus rhythm at 98 beats per minute, right axis deviation, complete right bundle branch block with a QRS duration of 130 ms; and an exclusive, 25 mm, R wave in V1 in favor of right ventricular hypertrophy

**Fig. 2:**
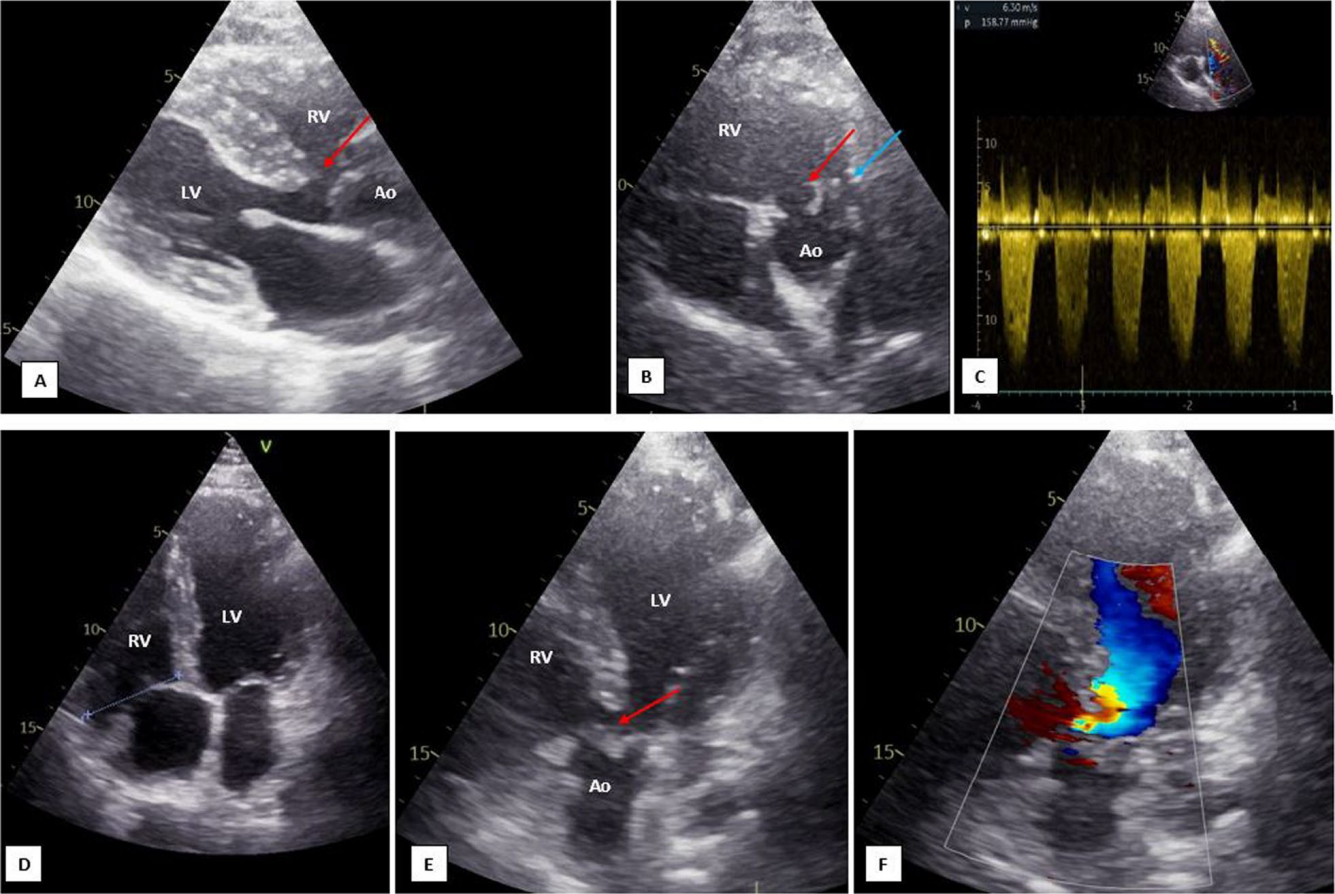
Transthoracic echocardiographic examination: **A** Parasternal long axis view illustrating a large malalignment-type VSD (red arrow), and an aorta overlapping the interventricular septum. **B** Parasternal short axis view illustrating the VSD (red arrow) and a hypoplastic main pulmonary artery (blue arrow). **C** Continuous Doppler objecting severe right ventricular outflow tract stenoses (*V*_max_ = 6.3 m/s). **D** Apical four chamber view illustrating a moderately dilated right ventricular (basal diameter = 43 mm). **E** Apical 5 chamber view illustrating the VSD (red arrow) and the overlapped aorta. **F** Color Doppler objecting bidirectional flow across the VSD. Ao: Aorta; LV: left ventricular; RV: right ventricular; and VSD: ventricular septal defect

The electrocardiogram showed regular sinus rhythm, right axis deviation, complete right bundle branch block with a QRS duration of 130 ms, and a 25 mm R wave in V1 without an S wave in favor of right ventricular hypertrophy (Fig. 1). The chest X-ray showed a cardiothoracic index of 0,48, a right ventricle enlargement with supra-diaphragmatic heart point, and a concave pulmonary arterial segment. The pulmonary vascularization was normal. Transthoracic echocardiography (TTE) diagnosed a TOF (Fig. 2, Additional file. 1, 2, 3). TTE showed a large outlet ventricular septal defect (VSD) of 16 mm with a bidirectional VSD shunt. The left ventricle was hypertrophied with normal function. The right ventricle (RV) was hypertrophied and moderately dilated with borderline systolic function (TAPSE = 17 mm, S' = 10 cm/s). The aorta was overriding the interventricular septum by 50%; it was not dilated with a mild regurgitation. The aorto-mitral continuity was preserved. The pulmonary stenosis was mainly valvular with severe main pulmonary artery hypoplasia (*V*_max_ = 6.3 m/s). TTE revealed a right aortic arch as well. Assessment of the pulmonary artery branches and coronary arteries was limited. We did not visualize an arterial duct or major aortopulmonary collateral arteries.

**Fig. 3:**
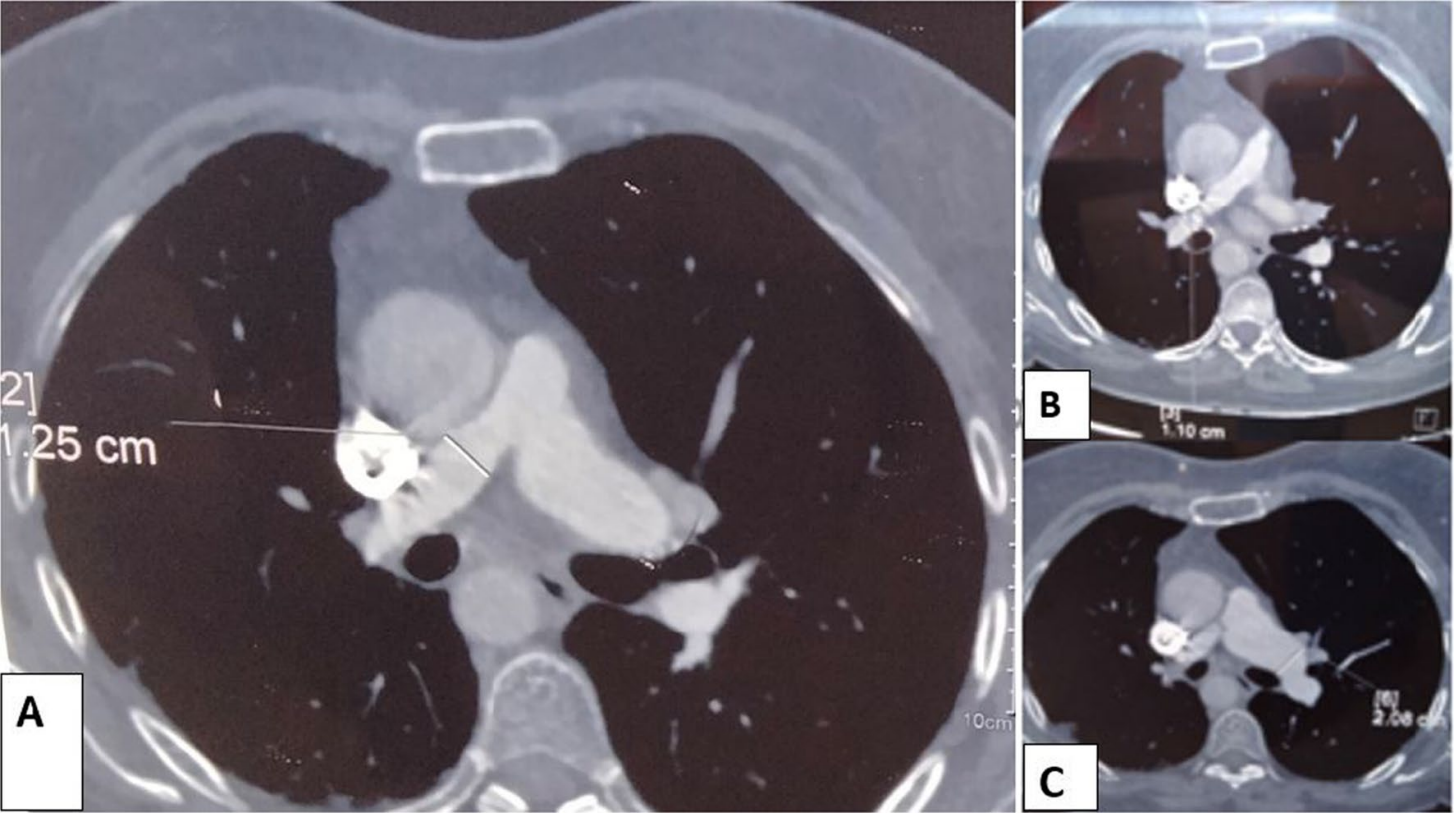
The dimensions of the pulmonary branches proximally (**A**) and peripherally (**B** and **C**) on the CT angiography of the chest

We repeated the CT angiography at our institution to better understand the pulmonary branches and the coronary arteries and search for an extracardiac shunt (Fig. 3). The CT angiography showed hypoplastic main pulmonary artery (with an ascending aortic/pulmonary trunk ratio of 2.5), a 29 mm large left branch, and a 13 mm large right branch. The aortic arch was to the right. The coronary arteries were normal, and no extracardiac shunt was seen. 24-h Holter monitoring revealed a permanent sinus rhythm with isolated atrial and ventricular extrasystoles. The patient refused to undergo further investigations and refused surgical treatment. A multidisciplinary discussion was undertaken, and the decision was to follow-up the patient clinically. There was a conundrum regarding the antihypertensive medications that should be employed in this circumstance. Vasodilators are usually avoided in situations of stenotic lesions. Beta-blockers were the best option in this case. But because the patient was well controlled on ACE inhibitors and she preferred to keep her treatment, we approved her choice.

## Discussion

The natural history of TOF is poor; the majority of patients die during childhood. The most common causes of death are hypoxic spells and cerebrovascular accidents during the first years of life and brain abscesses in older patients [4]. Only 3% of patients survive beyond the age of 40 [3]. A few cases of unrepaired TOF survivors are reported in the literature. The first case reported dates back to a 60-year-old musician in 1929 [5]. The oldest documented unrepaired TOF survivor is an 87-year-old woman [6].

Clinical presentation in survivors with uncorrected TOF was variable. Congestive cardiac failure was the clinical presentation in an 85-year-old man [7] and a 67-year-old man [8]. Nonspecific chest pain with dyspnea was the presenting symptoms in a 47-year-old man [9]. Our patient was paucisymptomatic, and the diagnosis was made incidentally. Unlike our patient, most of the reported patients with unoperated TOF were aware of the disease, and they refused surgical intervention earlier [7, 10]. It is worth noticing that only a few patients with TOF, estimated at 5% in one series [11], do not develop central cyanosis, as is the case with our patient.

Arterial hypertension was present in our case. It was also reported in two patients with unrepaired TOF aged over 60 years [8, 10]. ACE inhibitors were prescribed in both cases [8, 10]. Our case is unique since our patient underwent total thyroidectomy with no postoperative complications, and she delivered four times with no complications. Pregnancy is considered dangerous for unrepaired patients with TOF [12]. Variations in blood volume, a decrease in systemic peripheral resistance, and an increase in the right-to-left shunt are risk factors for poor tolerance during pregnancy. Delivery is equally a potential danger for these patients. In addition to maternal risks, fetal complications such as congenital defects and prematurity were reported [12]. Presbitero et al. revealed that, in patients with cyanotic CHD, the degree of cyanosis was the major risk factor for adverse fetal outcome [13].

At the age of 56, our patient maintained a sinus rhythm. Elderly age is a known risk factor for atrial fibrillation; nevertheless, unrepaired TOF undoubtedly increases its incidence [7, 14, 15].

As our case illustrates, TTE is the key examination in the diagnosis of TOF, even in elderly patients. Unlike the majority of reported cases [8], RV function was preserved in our patient. CT angiography revealed a caliber disparity between the pulmonary branches in our patient, which has not been previously reported in elderly patients with uncorrected TOF, according to our knowledge. The right aortic arch, a common anomaly associated with TOF [15, 16], was present in our patient.

In unrepaired TOF adult survivors, three main common features were reported [15]. Firstly, hypoplastic pulmonary artery with progressive development of infundibular stenosis, which was the presumed main reason in our patient. Secondly, the late development of left ventricle hypertrophy (LVH) might contribute to counteracting the amount of right-to-left shunt, as seen in our patient. Thirdly, the presence of an extracardiac shunt improving pulmonary perfusion such as systemic-pulmonary artery collaterals. Thoracic major aortopulmonary collateral arteries, but also abdominal major aortopulmonary collateral arteries were described in a 44-year-old survivor TOF [16]. Echocardiographic and CT examinations did not document an extracardiac shunt in our patient. The difficulty of maintaining such a precarious hemodynamic balance explains the rarity of this CHD in the adult population. Our patient remained paucisymptomatic until this age with no history of cyanotic spells because the pulmonary stenosis was mainly valvular. It is important to highlight that the natural history of TOF is always burdened with the risk of RV dysfunction evolving to biventricular heart failure and multiple organ failure. It is not sure how much longer our patient’s RV will tolerate the high pressure. The onset of atrial fibrillation may be a turning point in the natural history of the disease [15].

Surgical repair in patients aged 40 years or older is feasible, with a demonstrated improvement in functional status, but with increased operative risk, reduced survival, and significant re-intervention rate [17]. Since she was paucisymptomatic, our patient refused to undergo surgery. Regarding arterial hypertension management, we have considered angiotensin-converting enzyme inhibitors as a good therapeutic choice.

## Conclusions

Despite its rarity, patients with TOF can reach older ages without surgical correction. Our case highlights the divergence of the clinical presentation at this age. Late surgical repair should be meticulously decided on a case basis.

## Supplementary Information

**Additional file 1. Video 1:** Apical 5 chamber view illustrating the large VSD and the aorta overriding the interventricular septum. VSD: ventricular septal defect.

**Additional file 2. Video 2:** Parasternal long axis view illustrating the left ventricular hypertrophy and the anterior malalignement type VSD with color doppler objecting bidirectional flow across the VSD. VSD: ventricular septal defect.

**Additional file 3. Video 3:** Paraseternal short axis view illustrating the large outlet VSD and the severe main pulmonary artery hypoplasia. VSD: ventricular septal defect.

## Data Availability

No datasets were generated or analyzed during the current study.

## Abbreviations

ACE: Angiotensin-converting enzyme
CHD: Congenital heart disease
RV: Right ventricular
TOF: Tetralogy of Fallot
TTE: Transthoracic echocardiography
VSD: Ventricular septal defect
